# Mechanical and Early Long-Term Property Assessment of Foamed 3D-Printable Geopolymer Composite

**DOI:** 10.3390/ma18122837

**Published:** 2025-06-16

**Authors:** Rihards Gailitis, Liga Radina, Leonids Pakrastins, Andina Sprince

**Affiliations:** 1 Institute of High-Performance Materials and Structures, Riga Technical University, Kipsalas 6A, LV-1048 Riga, Latvia; 2 Institute of Civil Engineering, Riga Technical University, Kipsalas 6A, LV-1048 Riga, Latvia; liga.radina@rtu.lv (L.R.); leonids.pakrastins@rtu.lv (L.P.); andina.sprince@rtu.lv (A.S.)

**Keywords:** foamed geopolymer composites, fly ash-based geopolymer, 3D-printed geopolymer composites, long-term and mechanical properties

## Abstract

Additive manufacturing has been of considerable interest for the last 10 years. Cementitious composites have been developed to ensure fast and effective structure printing. To address sustainability and reduce the environmental impact of Portland cement-based composites, geopolymer composites have been developed that can be printed. This brings us to this study’s aim, which is to allow the printing of recycled lightweight structures with not only the ability to act as a structural material but also insulation capabilities. This study focuses on mix design development and the mechanical strength, creep, and shrinkage properties of these composites. The results show that foamed 3D-printed fly ash-based geopolymer composites may have reduced compressive strength, but still have sufficient strength to be used as a structural material. Furthermore, their creep and shrinkage strain are lower than those of the composite without foaming agent introduction.

## 1. Introduction

Additive manufacturing, or in other words, 3D printing, is increasing in popularity in civil engineering applications. It is claimed that by applying 3D printing methods in construction work, building costs can be reduced by up to 60% [1]. Three-dimensionally-printable concrete mixes are mainly tailored for specific printing techniques and even 3D printers. One thing that is common in 3D-printable cement composites is an increased amount of cement in the mix in contrast to similar composites meant for monolithic structures and on-site applications [2]. Printable cementitious composites are already made and available for mass usage, which further increases global cement consumption, leading to increased CO_2_ emissions. As CO_2_ emissions are still an important challenge that has to be overcome to further resolve climate change, there is high demand for alternative printable cementitious materials with reduced CO_2_ footprints that retain the same or only slightly reduced mechanical and long-term properties. The possible solution to reducing CO_2_ is a geopolymer matrix containing cementitious material. When life cycle assessments were performed on geopolymer concretes (GCs) and they were compared to Portland cement concretes [3], the main findings showed that not all composites of GC have environmental benefits in regard to their created emissions. For instance, rice husk- and metakaolin-based geopolymers show higher greenhouse gas emissions than Portland cement concrete. The same is true for NO_x_ emissions. Geopolymer composites based on fly ash (FA) and ground granulated blast furnace slag (GGBFS) have significantly lower CO_2_ and NO_x_ emissions. In this regard, it has to be mentioned that fly ash-based GP concretes cost more than twice as much as PC concretes. Thus, it was mentioned by [4] that FA and GGBFS geopolymer concretes have lower embodied energy than Portland cement concrete, mainly due to the fact that they do not require additional processes for the preparation of their constituents for use as a binder.

The field of civil engineering is rapidly developing, and to meet the demand for not only housing but also infrastructure, which is increasing year-on-year, it is of high importance to not only develop and improve 3D printing capacity but also to adapt and create materials that will have a smaller burden on the environment. It has been estimated that 3D printing can reduce waste created by construction by 30 to 60%. Furthermore, by using 3D printing techniques, production times will be decreased by 50 to 70%, and labor costs will drop by 50 to 80% [5]. It has been claimed and proven that in certain load application cases, the weakest link is the interlayer connection zone of printed specimens. Specimens also exhibit anisotropic behavior and insufficient insulating properties, which lead to possible heat loss [6,7]. There are several techniques for creating 3D-printed structures. The main and most common ones are based on extrusion and powder bed systems [8]. Both of these techniques have challenges regarding the foamed materials. An extrusion system, while extruding, does compact the materials, therefore squeezing out pores and air entrapment that has been created by the introduction of the foaming agent. A powder bed system would have significant difficulty with the foaming of the layer while an additional layer is created on top of the previous one, creating an unstable system.

Two main challenges posed by the chosen material type and creation method are the ability to hold the shape of the material after extrusion and the ability to support the above extruded material without significant deformation [9]. Here a huge role is played by the mixing sequence and chemical composition of the geopolymer composite.

This article is devoted to developing fly ash-based 3D-printable geopolymer composites that are foamed and are not only suitable for use as a structural material but also have some insulating capabilities. In this study, hydrogen peroxide is used as the foaming agent.

## 2. Materials and Methods

### 2.1. Development of Printable Geopolymer Composite

The first challenge in regard to this study was to choose a geopolymer composition that is not fast-setting, and with a dense consistency, so that it can be printed using an extruding 3D printer. A composition was chosen as a base point that has been previously used in the creation of cast geopolymer composites and was based on class F fly ash. This specific fly ash was sourced from Skawina (Poland). It has spherical aluminosilicate particles. It is rich in oxides such as SiO_2_ (47.81%) and Al_2_O_3_ (22.80%). The composition of the initial printed composite, as well as the alkali solution composition, are shown in Table 1.

**Figure 1:**
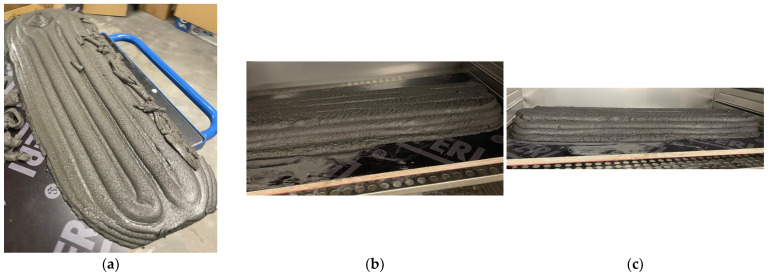
Initial printing results of Original REF (**a**), REF with coarser filler (**b**), and REF with addition of slag (**c**).

The composition of the mix turned out to be slow-setting in ambient laboratory conditions and had self-leveling or non-Newtonian fluid properties, thus leading to insufficient layer-holding abilities and inapplicability for further usage without modifications. The initial printing results are shown in Figure 1a.

The main assumption from the literature review was that, first of all, alkali solution has the ability to act as a plastificator, and also, when it dissolves in the solution, fly ash acts as a wheel bearing and reduces inner friction, leading to a self-leveling effect. Because of these two main challenges, we focused on the following: 1. how to make a printed composite hold its layers when multiple layers have been put on top of it; 2. how to make a composite partially set prior to heat treatment introduction for full polymerization.

To partially resolve the previously mentioned challenges, GGBFS was chosen to be added in small amounts (to 20% of the matrix material amount). The main reason was to mimic the cement composite mix design, and the second reason pertained to the particle characteristics of the GGBFS—they are edgy with uneven surfaces, creating increased inner friction as well as creating early setting of the composite. Table 2 shows the compositions that we tried, but it was found that the effect was not satisfactory.

It was observed that while the composite printed significantly better than previous ones and held the layers’ weight rather well, the biggest issue was the matrix, or lack of it. When the printed elements were cut into prismatic specimens (40 × 40 × 160 mm), it was visible that filler (sand) grains were easily coming off the specimens.

**Figure 2:**
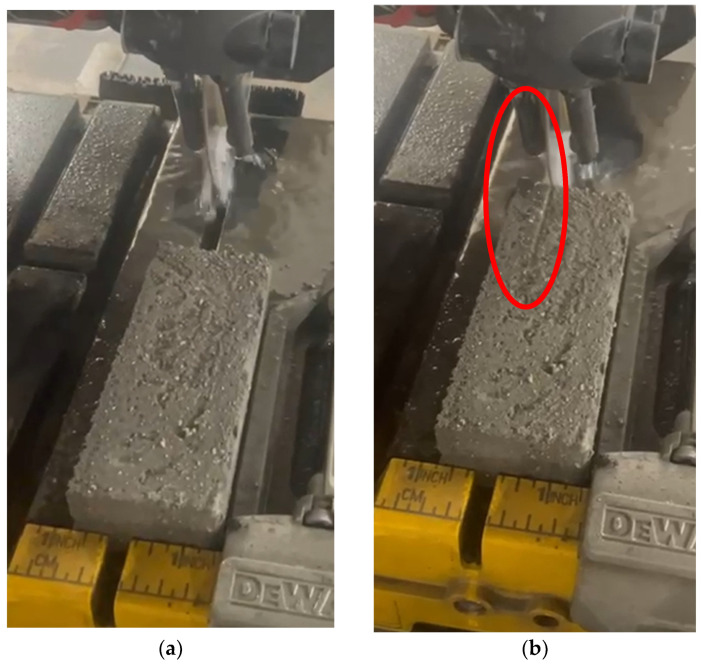
Three-dimensionally printed geopolymer composites (REF with slag) cutting (**a**) and resistance to water jetting on the surface of it (the red circle) (**b**).

When the specimens were dried in a heat chamber (35 °C for 24 h), the sand grain detachment from the specimens was unsatisfactory (as shown in Figure 2). Mechanical tests were performed, but no significant resistance to the force applied by the compressive strength press was exhibited. Therefore, based on these unsatisfactory results, the decision was made that the setting of the printed composites had to be increased. For this purpose, a small amount of Aalborg cement was chosen (2% by weight of the dry components). The composition of the end composite for printing is shown in Table 3.

The whole process of creating this foamed geopolymer composite can be divided into three parts:

Dissolution:

$${\mathrm{Al}}_{2}O_{3}\cdot {\mathrm{xSiO}}_{2}\left(\mathrm{from}\;\mathrm{fly}\;\mathrm{ash}\right)+{\mathrm{OH}}^{-}\to \mathrm{Al}{\left(\mathrm{OH}\right)}_{4}^{-}+\mathrm{Si}{\left(\mathrm{OH}\right)}_{4}$$

Polycondensation:

$$\mathrm{Si}{\left(\mathrm{OH}\right)}_{4}+\mathrm{Al}{\left(\mathrm{OH}\right)}_{4}^{-}+{\mathrm{Na}}^{+}+{\mathrm{extra}\;\mathrm{Si}\;\mathrm{from}\;\mathrm{Na}}_{2}{\mathrm{SiO}}_{3}\to \mathrm{Nan}\left[-{\left({\mathrm{SiO}}_{2}\right)}_{z}{\left({\mathrm{AlO}}_{2}\right)}_{n}\right]\cdot {\mathrm{wH}}_{2}O$$

Foaming reaction:

$$H_{2}O_{2}\overset{\mathrm{alkaline}}{\to }H_{2}O+\frac{1}{2}O_{2}↑$$

As a result, a printable composite was obtained. It was observed that this composite is rather active in terms of setting. Also, after initial printing and polymerization in the heat chamber, both specimens with (Mark II) and without (Mark I) Aalborg cement addition were observed under a scanning electron microscope (SEM). As a result, it was determined that the mixing stages and the time in between are very crucial to allow fly ash particles to properly dissolve. As a result, the mixing sequence was changed as follows: the 1st dry mix, except Aalborg cement (fly ash, GGBFS, sand), was mixed on low speed (equal to 100 revolutions per minute (rpm)) for 2 min to obtain a homogeneous dry mix. Afterwards, an alkali solution was added as well as half of the water, and the whole mixture was mixed for 8 min with varying mixing speeds. High-speed mixing was started at 288 rpm for 30 s to 1 min to prevent spilling of the solution and water; the speed was gradually increased after one minute to 385 rpm and maintained for 2 min, and finally increased to 500 rpm for 5 min. Then, the mixed geopolymer composites were left to settle for at least 30 min (mostly 45 min, but 30 min allowed the fly ash to dissolve to an acceptable level as well). After the initial consolidation time, the composition was remixed by itself for a minute, and Aalborg cement was added with the remaining water. This whole composition was mixed for an additional 2 to 3 min at a high mixing speed and then introduced to the 3D printer chamber. The extrusion of the mixture started right after, and a beam-shaped specimen with four layers horizontally and four layers vertically was extruded over the next 5 min. Then, 15 to 20 min after introducing cement into the mix, the mixture had gained significant stiffness and could structurally hold itself.

**Figure 3:**
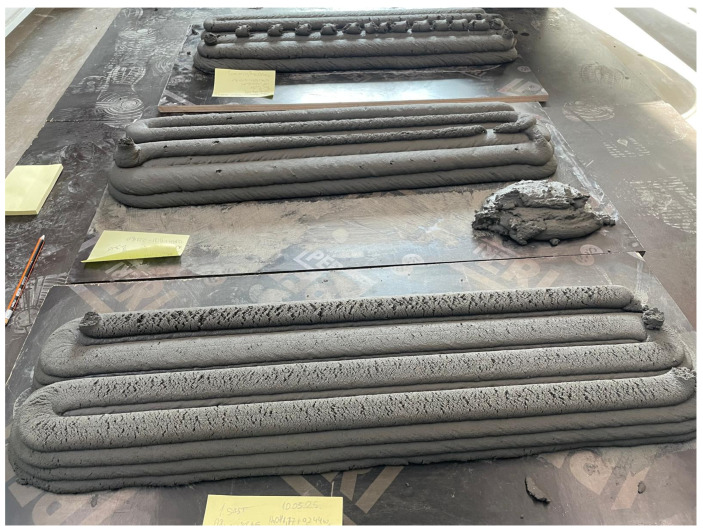
Three-dimensional printing results of composition detailed in Table 3 starting from top to bottom: Optimized REF, 1% H_2_O_2_, and 2% H_2_O_2_.

As is apparent from Figure 3, the end results of the printed composites were favorable not only for the unfoamed specimens but also for the foamed specimens.

### 2.2. Mechanical Property Assessment

After composite printing, and initial setting and hardening in a heat chamber, samples were cut into 40 × 40 × 160 mm prismatic specimens. When the specimens were cut, an initial assessment of their composition suitability and potential load-bearing ability was performed. In this case, during cutting, several composites were damaged by the water jetting of the concrete saw blade. Based on this, it was determined that the matrix is not capable of holding together the whole composite, or there is an insufficient amount in the specimen, and some or a significant amount of filler is not connected. After the specimen were cut, they were dried in a heat chamber for 24 h at 35 °C to take out excess moisture caused by cutting. Then, the specimens were subjected to compression tests with a hydraulic press. The press was operated at a loading speed of 0.8 MPa/s.

### 2.3. Preparation of Creep and Shrinkage Specimens

**Figure 4:**
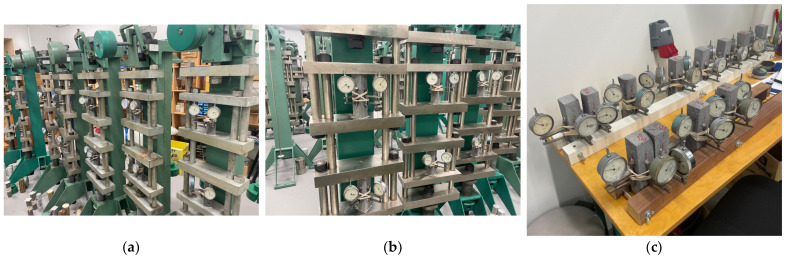
Creep test (**a**,**b**) of 3D-printed foamed and unfoamed specimens and shrinkage test of these specimens (**c**).

When the printable composites and their mechanical strength had been determined, suitable compositions were determined for long-term testing purposes. First of all, the specimens were shaped in the same way as the ones intended for mechanical property determination. Creep and shrinkage tests were carried out for 28 days, and the tests were based on RILEM TC 107 [10]. To connect and record the deformation of the specimens, aluminum plates were glued using epoxy resin on two surfaces of the specimens. After 24 h of glue setting, rubber bands were tied around the specimens, and dial gauges were attached with a measuring base of 50 mm. The specimens were placed in creep lever test stands, and simultaneously, separate specimens were placed near the creep rigs in the same room to record drying shrinkage. The laboratory conditions for the creep and shrinkage tests were 24 ± 1 °C and 30 ± 3% relative humidity. The creep and shrinkage setups are shown in Figure 4.

## 3. Results and Discussion

For the determination of suitable compositions of the prepared geopolymer composites that could have some load-bearing capacity and the capability of foaming, they were selected based primarily on their printability, but also with regard to their load-bearing capacity. The first specimens, which were based on an optimized cast geopolymer composite composition, did have favorable printability, but when the samples were cut into prismatic specimens, they appeared to have an insufficient amount of matrix or an insufficient level of polymerization. To verify and support these claims, samples from the specimens were taken and examined under a scanning electron microscope (SEM).

**Figure 5:**
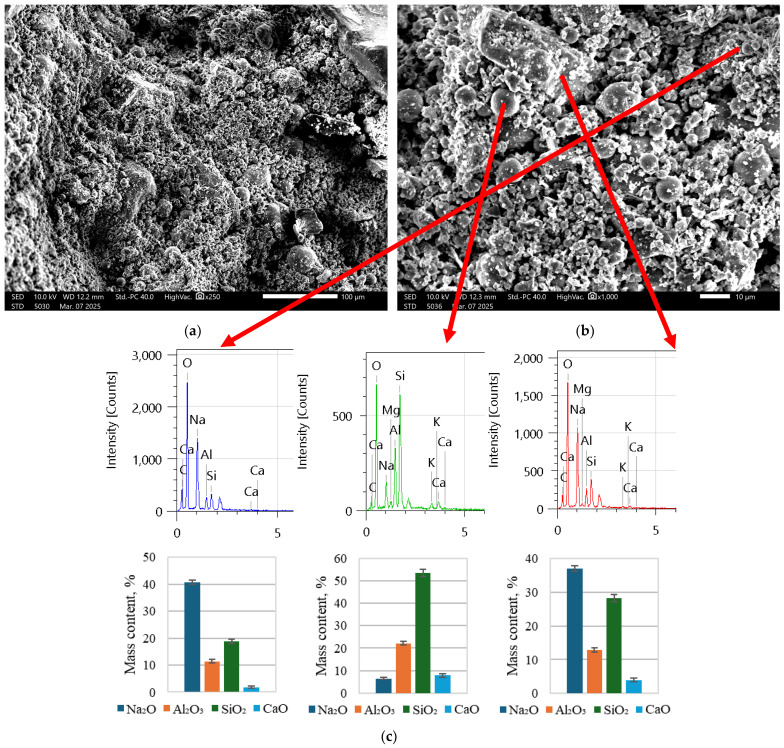
Samples of REF specimens (Table 2) at (**a**) 250 times magnification and (**b**) 1000 times magnification; (**c**) EDX spectra of chemical compositions at specific points and corresponding main oxide content descriptions.

**Figure 6:**
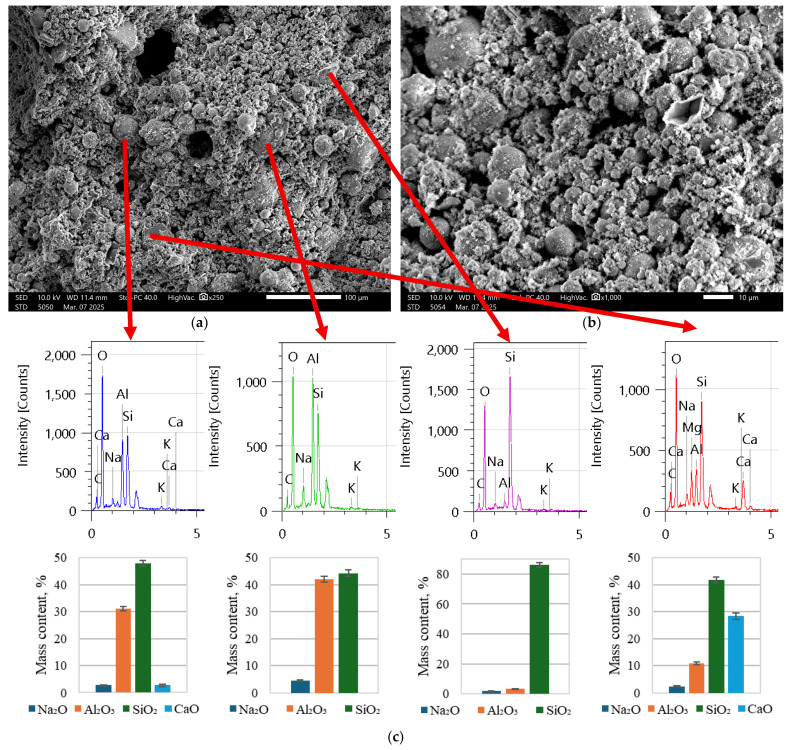
Sample of REF with slag (foamed) specimens (Table 2) at (**a**) 250 times magnification and (**b**) 1000 times magnification; (**c**) EDX spectra of chemical compositions at specific points and corresponding main oxide content descriptions.

**Figure 7:**
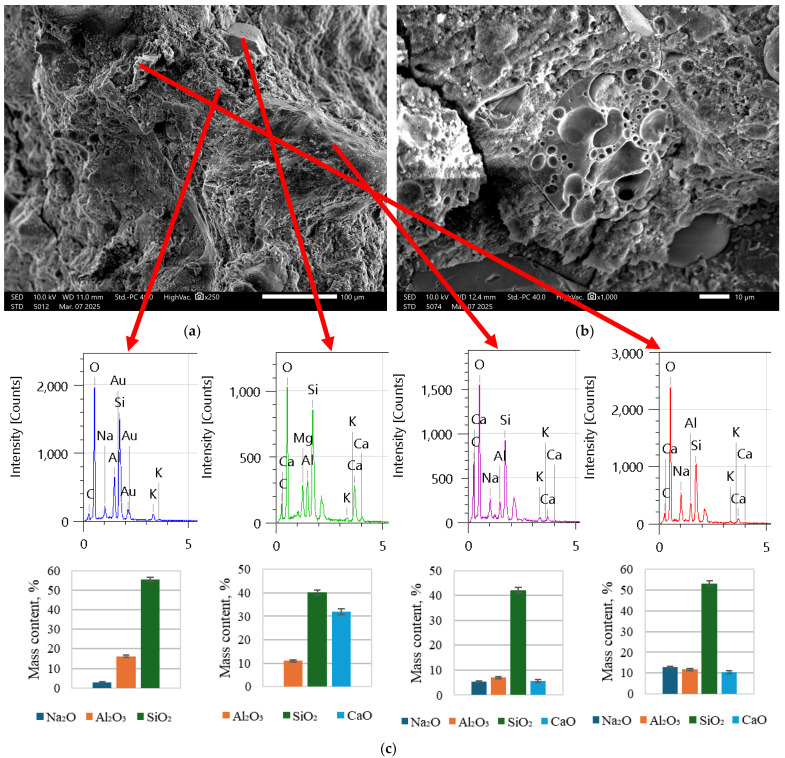
REF (Table 3) specimens under SEM at (**a**) 250 times magnification and (**b**) 1000 times magnification; (**c**) EDX spectra of chemical compositions at specific points and corresponding main oxide content descriptions.

As is visible in Figure 5, there is a significant number of fly ash particles, as is also visible in Figure 6. In Figure 7, the structure is a much more solid structure with only a couple of unreacted fly ash particles.

It was determined based on Figure 5 and Figure 6 that composites have to be premixed with mostly alkali solution together with water to initiate the dissolving of fly ash in order to create a suitable matrix for long-term strength (after polymerization). As is clear from Figure 7, the number of spherical particles (fly ash particles) is significantly less than in Figure 5 and Figure 6. Furthermore, by introducing cement with a little bit of water (the amount necessary for hydration and workability), the printed composite directly exhibits a rigit structure. If the EDX spectra and oxide quantitative analysis are analyzed, it is clear that in the specimens in Figure 5 and Figure 6, there is a significant amount of unreacted Na and Al, while in Figure 7c, most of the Al has had the desired reaction, and the amounts are rather low. In both cases, we can see unreacted CaO that corresponds to added Portland cement. Based on these results, for further printed composite preparation, this technique was used as described in the above paragraph.

### 3.1. Assessed Mechanical Property

**Figure 8:**
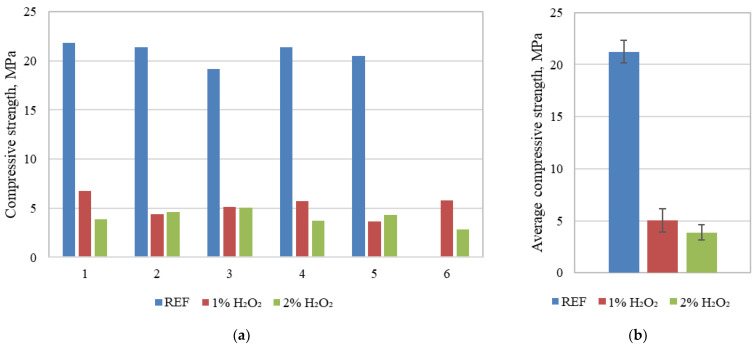
Compressive strength of the tested foamed and unfoamed 3D-printed specimens (**a**) and average compressive strength with standard deviation (**b**).

According to the techniques and procedure explained in Section 2.2, the compressive strength values were determined. The resulting compressive strength of the specimens is shown in Figure 8.

While it is clear that foaming agents have significantly reduced compressive strength, their strength is still high enough for them to be considered as a structural material that can be used to create load-bearing structures. In fact, the compressive strength reduces, on average, by 76.17% when 1% of foaming agent is introduced, and by 81.72% when 2% of foaming agent is introduced. The standard deviation of the compressive strength results for the reference and 1% foamed specimens is the same, while for 2% foamed specimens, the standard deviation is 30% lower. The density of the specimens when 1% foaming agent is introduced reduces by 21.20%, but when specimens are foamed with 2% foaming agent, the density reduces by 29.18%, in contrast to the reference specimens without foaming agent. If we look at previously created printed cementitious composite specimens [11], then it is visible, first of all, that geopolymer specimens exhibit higher strength (on average, more than two times more). Even the foaming of a geopolymer composite does not reduce its strength so significantly. In contrast to the cementitious composite, the 1% H_2_O_2_ foamed geopolymer composite’s strength is 37.5% lower, and that of the 2% H_2_O_2_ composite is 51.5% lower.

### 3.2. Long-Term Properties

Using the previously mentioned compressive strength values, specimens intended for the creep test were loaded with 20% of their compressive strength value. The test was carried out for 28 days.

**Figure 9:**
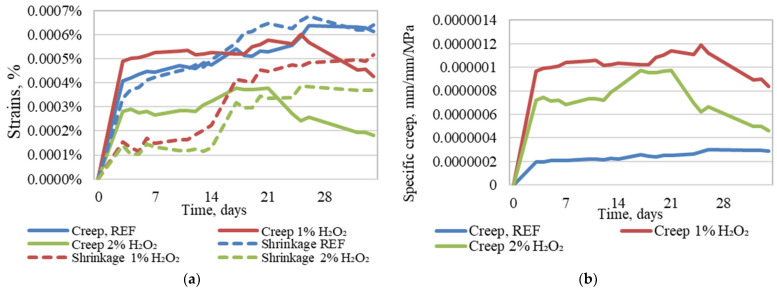
Recorded creep and shrinkage strain (**a**) and specific creep (**b**) of unfoamed as well as 1% H_2_O_2_ and 2% H_2_O_2_ foamed printed geopolymer specimens.

When the creep strain is acknowledged (in Figure 9a) in the specimens, it may appear that specimens without a foaming agent, as well as those with 1% foaming agent inclusion, have the largest and therefore the most unsatisfactory properties of the tested printed geopolymer composites. Furthermore, specimens with no foaming exhibit the largest shrinkage strain, while 1% H_2_O_2_ specimens, on average, have 17% lower creep and 42% lower shrinkage strain, while 2% H_2_O_2_ specimens exhibit 45% lower creep strain and 57% lower shrinkage strain.

Taking into consideration that each type of printed composite specimens is subjected to different load values that correspond to the same stress level for all of the tested geopolymer composite compositions, the specific creep values (Figure 9b) are calculated according to Equation (1), and it is also shown that the strain amount does not totally reflect the specimens’ willingness to creep.

(1) $$\chi_{cr}\left(t,t_{0}\right)=\frac{\varepsilon_{cr}\left(t,t_{0}\right)}{\sigma }=\frac{\varepsilon_{kop}\left(t\right)-\varepsilon_{sh}\left(t\right)-\varepsilon_{el}\left(t,t_{0}\right)}{\sigma }=\frac{1}{E_{cr}\left(t,t_{0}\right)}$$

The following are used in the formula: $\chi_{cr}\left(t,t_{0}\right)$ is the specific creep; $\varepsilon_{cr}\left(t,t_{0}\right)$ is the creep strain; $\varepsilon_{kop}\left(t\right)$ is the total strain; $\varepsilon_{sh}\left(t\right)$ is the shrinkage strain; $\varepsilon_{el}\left(t,t_{0}\right)$ is the elastic strain; σ is the compressive stress; $E_{cr}\left(t,t_{0}\right)$ is the modulus of creep.

From these curves it is clear that as well as compressive strength being significantly reduced by geopolymer foaming, willingness to creep is also significantly increased, while the specimens without foaming have a steady specific creep level that, on average, is 76% lower than with 1% H_2_O_2_ foamed specimens, and 63% lower than specimens foamed with 2% of H_2_O_2_.

## 4. Conclusions

Following the developed and tested geopolymer composition, the following conclusions can be drawn:

1. To obtain a 3D-printable geopolymer composite, it is necessary to incorporate small amounts of blast furnace slag and Portland cement. This is due to the necessity for slight setting of the printed layers of the material to support layers printed on top.
2. Hydrogen peroxide’s usage as a foaming agent and incorporation into 3D-printed geopolymer composite mix does reduce the compressive strength of the printed specimens by 76.17% and 81.72% with 1% and 2% foaming, respectively. Nevertheless, the compressive strength of composites reduces to 3.88 MPa for specimens with 2% hydrogen peroxide, which is suitable for the creation of load-bearing structures for low-rise building.
3. Specimens without foaming agent introduction exhibit the largest creep and shrinkage strain. While this raises concerns when the willingness to creep or specific creep is determined, the lowest specific creep values are for the specimens without a foaming agent. For specimens with 1% and 2% hydrogen peroxide inclusion, the specific creep is 76% and 63% higher, respectively.

## Figures and Tables

**Table 1:** Source composition of the printable geopolymer composite.

Alkali Solution		Mix Composition	
Constituent	Weight (g)	Constituent	Weight Ratio
NaOH flakes	400	Quartz sand	1.00
Water	1000	Fly ash	1.00
R-145 Na_2_O + SiO_2_ solution (molar module 2.5, density 1.45 g/cm^3^)	3500	Dry mix/solution ratio	0.01

**Table 2:** Initial optimization of the geopolymer composite intended for 3D printing.

Constituents	Original REF	REF with Coarser Filler	REF with Slag
	Mass, kg	Mass, kg	Mass, kg
Fly ash	3.60	3.60	3.60
GGBFS	-	-	0.80
Sand 2–8 mm	-	5.4	-
Sand 0–2 mm	10.8	5.4	8.00
Alkali solution	4.032	3.820	1.280
Water	-	-	1.468

**Table 3:** Printable geopolymer composite with addition of GGBFS and Aalborg cement.

Constituents	REF	1% H_2_O_2_	2% H_2_O_2_
	Mass, kg	Mass, kg	Mass, kg
Fly ash	3.60	3.60	3.60
GGBFS	0.80	0.80	0.80
Aalborg cement CEM I	0.24	0.24	0.24
Sand 0–2 mm	8.00	8.00	8.00
Alkali solution	2.014	2.014	2.014
Water	0.668	0.668	0.668
Hydrogen peroxide	-	0.124	0.248

## Data Availability

The original contributions presented in this study are included in the article. Further inquiries can be directed to the corresponding author.
